# Diagnostic ability of intraoperative ultrasound for identifying tumor residual in glioma surgery operation

**DOI:** 10.18632/oncotarget.20394

**Published:** 2017-08-22

**Authors:** Guangying Zhang, Zhanzhan Li, Daolin Si, Liangfang Shen

**Affiliations:** ^1^ Department of Oncology, Xiangya Hospital, Central South University, Changsha, Hunan Province 410008, China; ^2^ Department of Pediatric Neurology, Xiangya Hospital, Central South University, Changsha, Hunan Province 410008, China

**Keywords:** intraoperative ultrasound, glioma residual, diagnostic test, meta-analysis

## Abstract

Achieving total glioma resection represents a major challenge to neurosurgeons with no distinct margin between tumor and surrounding brain tissue. Many imaging methods are employed in surgery visualization and resection control. We performed this meta-analysis to assess the diagnosis value of intraoperative ultrasound and judged whether ultrasound is a suitable tool in detecting glioma residual. The databases including PubMed, Embase, Web of Science, China National Knowledge Infrastructure (CNKI), Wanfang and Weipu were systematically searched to find out relevant studies and published up to May 5, 2017. A total of 14 studies involving 542 participants met the selection criteria and bivariate mixed effects models were used for analysis. The parameters and their corresponding 95% confidence interval (CI) were computed on Stata 12.0 software. The pooled sensitivity was 0.75 (95%CI: 0.62–0.84), specificity was 0.88 (95%CI: 0.79–0.94), positive likelihood ratios was 6.27 (95%CI: 3.76–10.47), negative likelihood ratios was 0.29 (95%CI: 0.20–0.42), diagnostic odds ratios was 21.83 (95%CI: 14.20–33.55) and area under the curve of summary receiver operator characteristic was 0.89. Stratified meta-analysis showed sensitivity and area under the curve in low-grade glioma were both higher than high-grade glioma. The Deek's plot showed no significant publication bias (t = −1.03, *P* = 0.33). Intraoperative ultrasound has high overall diagnostic value to identify glioma remnants, especially in low-grade glioma, which shows a benefit for prognosis and life quality of patients. In general, Intraoperative ultrasound is an effective tool for maximizing the extent of glioma resection.

## INTRODUCTION

Glioma is the most common primary intracranial tumor account for about 40% of central nervous system tumors and 70% of malignant brain tumors [1], Whose treatment protocols are mostly based on surgical treatment, radiation therapy or temozolomide adjuvant chemotherapy [2]. However, the primary treatment of glioma is microscopic surgical resection, which must be maximally remove the tumor tissues and preserving normal nerve function. But it is a challenge to resect total tumor lesion due to the ambiguous boundary between tumor and normal tissue with glioma invasive growth [3–4]. Thus, the precise tumor localization and boundary identification are required to improve surgical strategies. The real-time images and position marks properties of intraoperative ultrasound (IOUS) were extensively used in tumor location, residue monitoring, guidance of aspiration biopsy and imaging blood flow of intracranial glioma [5–6], which is easy to operate and can be applied repeatedly. In comparison, intraoperative magnetic resonance imaging (iMRI) and navigational positioning system are limited by the expensive facility and need to avoid bias of brain transformation or shifting after skull opening in detecting intracranial glioma remnants[7–8]. In recent years, the clinical application of detection equipment for tumor residual may decrease the rate of malignant degeneration and prolong the median survival time and progression-free interval through the radical excision [9]. However, the diagnosis value of IOUS to detect the intracranial tumor removal is remains controversial due to the absence of cases and long-term follow-up data. And a point of ongoing discussion of iMRI, PET and navigational positioning system are superior to IOUS with insufficient data supporting. Thus, we performed this systematical review and meta-analysis to investigate the diagnostic values of IOUS in glioma operation.

## RESULTS

### Selection and characteristics of studies

**Figure 1:**
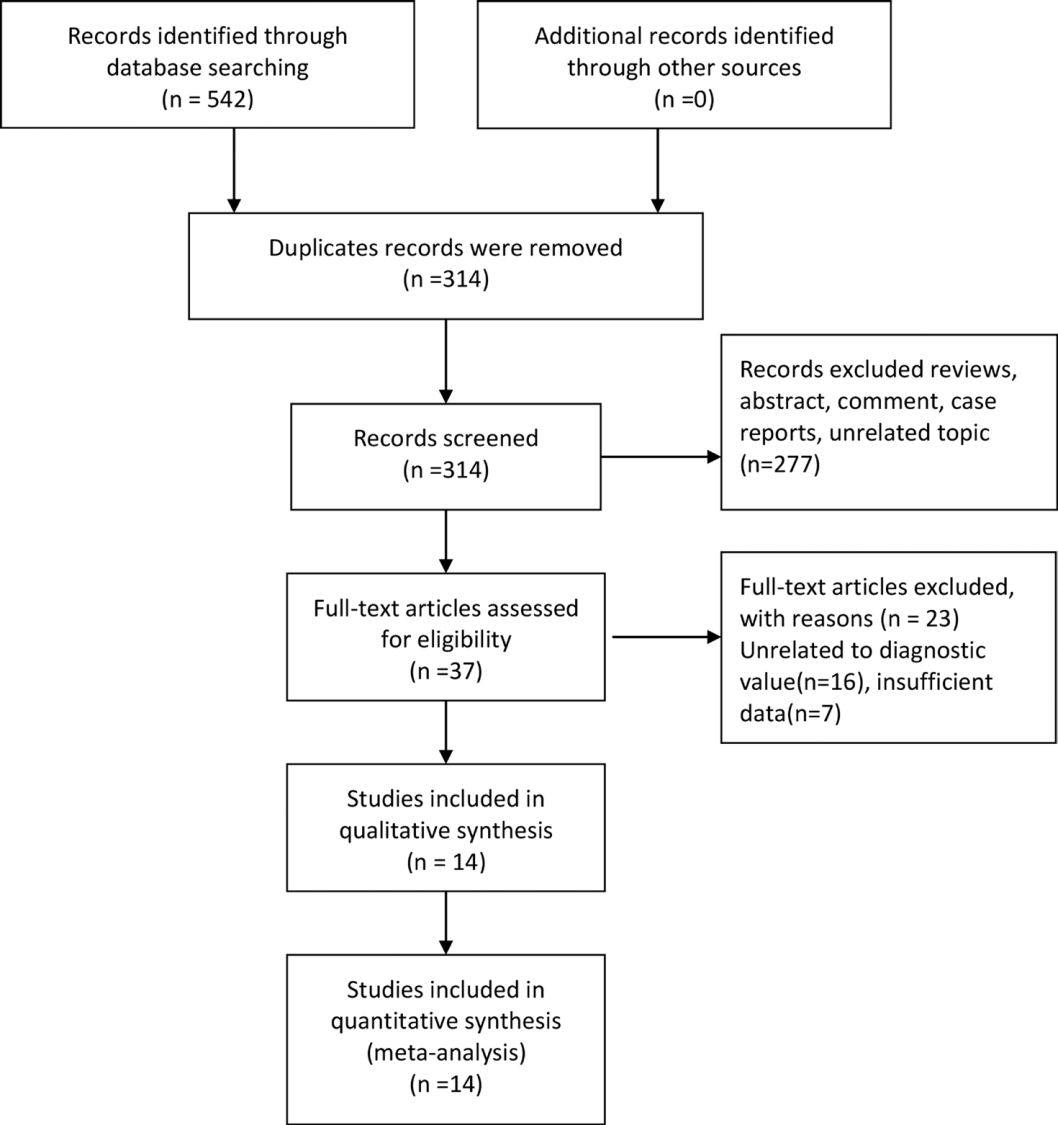
Flow diagram of studies selection process

A total of 542 articles were yielded in primary literature search, which included 147 records identified from PubMed, 36 records from Embase, 90 records from Web of Science, 106 records from China National Knowledge Infrastructure (CNKI), 88 records from Wanfang and 75 records from Weipu. After screening duplicate and unrelated topic, 37 articles were identified to further read by retrieving the full text. Finally, on the basis of above inclusion criteria, 14 articles were included with 1946 samples [10–14, 15–23]. The literature screening process and results are given in Figure 1.

The diagnostic accuracy of ultrasound for detecting glioma residual when the surgeons were considered excised completely should evaluate in all included studies. We know a patient could take more than one biopsy samples, only a few studies (*n* = 3) take patients as their object of study. When the patients and nidus on biopsy were both presented, we employed the latter, because the ultrasound results were one-to-one correspondence to every focus. We extracted the author, country, year of publication, mean age, sample size from the eligible articles, which were published between 1996 and 2016. The basic characteristics of the included studies are given in Table 1.

**Table 1:** Characteristics of the included studies in the meta-analysis

No.	Author	Year	Region	Mean age(y)	Sample size	Study design	Study population	TP	FP	FN	TN
1	He	2012	China	35.6 ± 8.2	38	Prospective	Single center	5	4	5	24
2	Qiu	2015	China	45.1 ± 13.1	173	Prospective	Single center	20	10	13	130
3	Yang	2014	China	43.47 ± 13.83	83	Prospective	Single center	13	10	8	52
4	Wang	2009	China	39.0 ± 10.9	150	Prospective	Single center	48	7	21	74
5	Tian	2009	China	41	189	Prospective	Single center	101	19	25	44
6	Guo	2011	China	41.6	373	Prospective	Single center	48	5	36	284
7	Chen	2007	China	39.6	216	Prospective	Single center	23	3	12	178
8	Liu	2009	China	22–68	80	Prospective	Single center	44	11	6	19
9	Woydt	1996	German	45.8	78	Prospective	Single center	47	6	6	19
10	Becker	1999	German	45.6	31	Prospective	Single center	24	2	1	4
11	Chacko	2003	India	38.2 ± 8.8	96	Prospective	Single center	66	13	2	15
12	Venelin	2011	German	-	11	Prospective	Single center	5	1	1	4
13	Shu	2016	China	39.6 ± 6.8	360	Prospective	Single center	69	18	42	231
14	Jan	2015	German	56	68	Prospective	Single center	12	1	37	18

### Quality evaluation

The summary of the quality assessment with regard to risk of bias and applicability concerns is shown in Supplementary Figure 1 and Supplementary Figure 2. We scored the questions as “low”, “high”, or “unclear” to examine included studies. As we can see, 1 study in patient selection is treated as high concern for applicability concerns. In summary, the quality of included studies met requirements for meta-analysis.

### Quantitative synthesis

**Figure 2:**
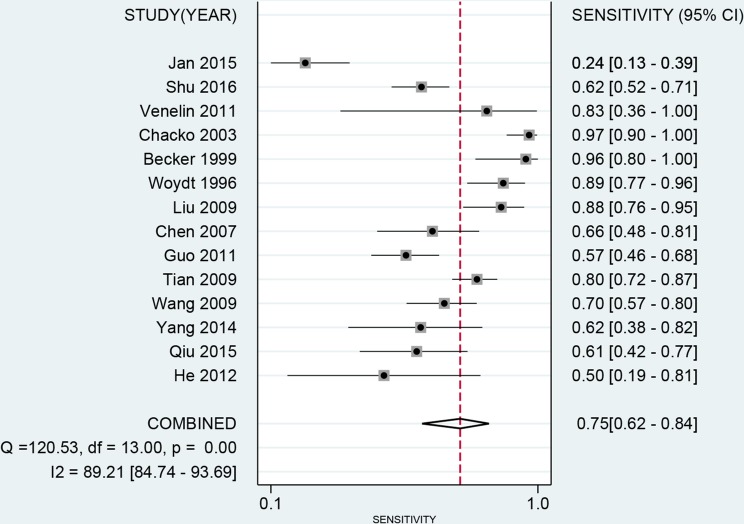
Forest plot of pooled sensitivity of intraoperative ultrasound for glioma residual

**Figure 3:**
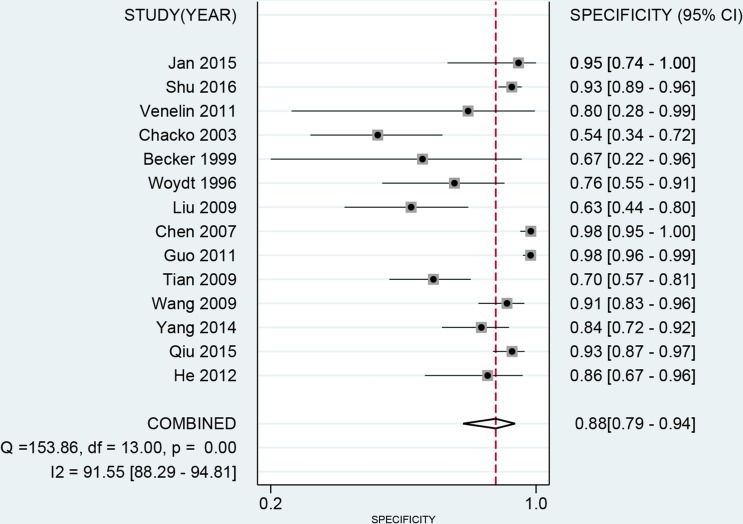
Forest plot of pooled specificity of intraoperative ultrasound for glioma residual

**Figure 4:**
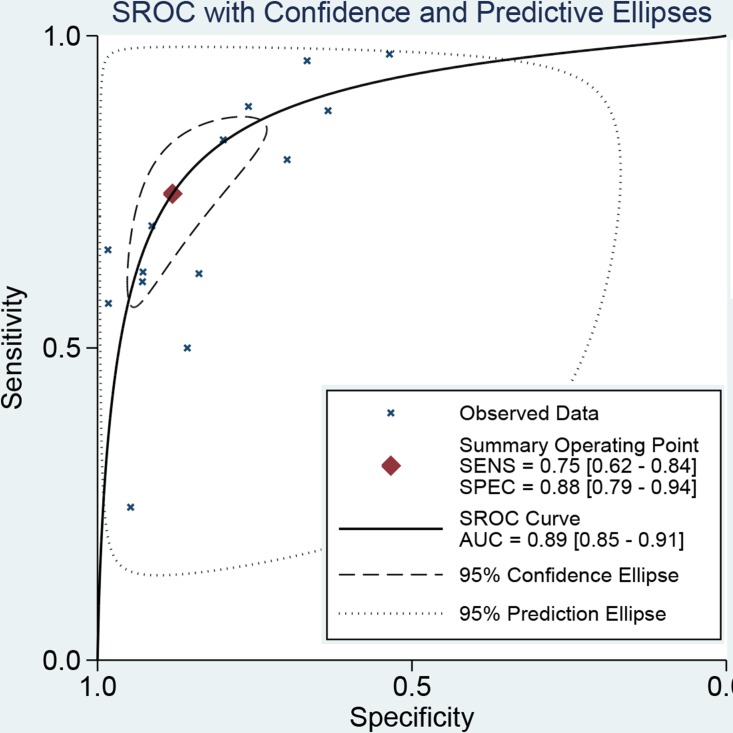
The SROC curve of intraoperative ultrasound for glioma residual

**Figure 5:**
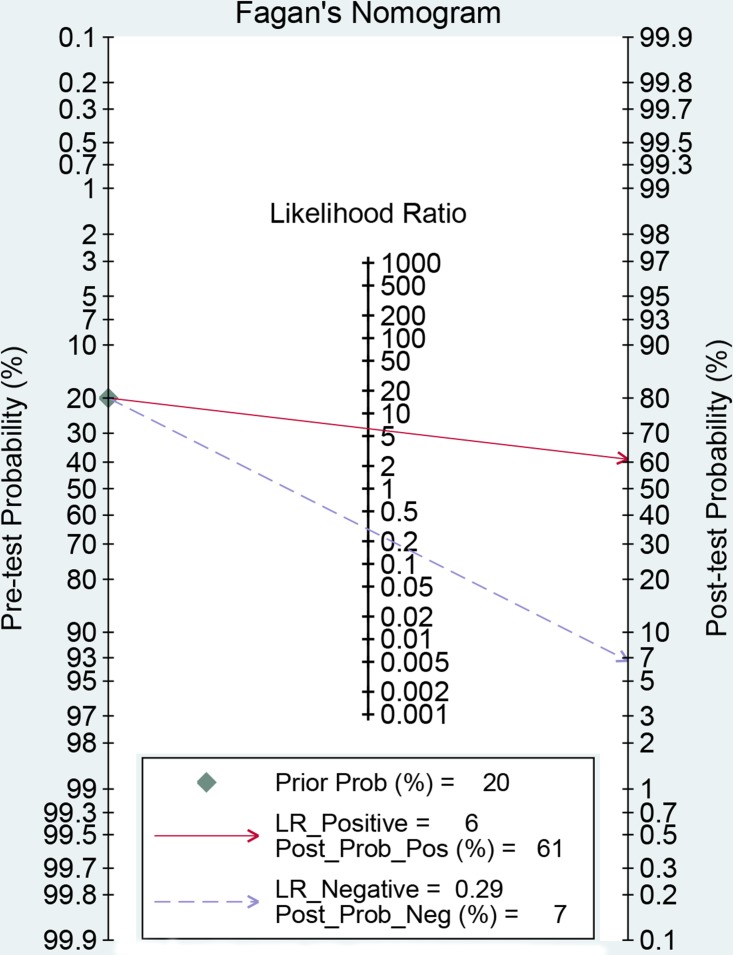
Fagan diagram evaluating the overall diagnostic value of intraoperative ultrasound for glioma residual

The latest Preferred Reporting Items for Systematic Reviews and Meta-Analyses Protocols were applied here. The study selection, data extraction, and risk of bias evaluation were performed by two authors independently to ensure the quality of this meta-analysis. Notable heterogeneity was observed on account of I^2^ = 89.21%, *P* < 0.01 for sensitivity and I^2^ = 91.55%, *P* < 0.01 for specificity. Therefore, a random effects model was adopted for this meta-analysis. We calculated the pooled sensitivity of 0.75 (95%CI: 0.62–0.84), pooled specificity of 0.88 (95%CI: 0.79–0.94). The overall positive likelihood ratios (PLR) and negative likelihood ratios (NLR) were 6.27 (95%CI: 3.76–10.47) and 0.29 (95%CI: 0.20–0.42), respectively. The diagnostic odds ratios (DOR) was 21.83 (95%CI: 14.20–33.55) (Figure 2 and Figure 3). Based on the sensitivity and specificity, we generated an summary receiver operator characteristic (SROC). The area under the curve (AUC) of the SROC was 0.89 (95%CI: 0.85–0.91), which suggested that IOUS has a relatively high diagnostic value for detecting glioma residual (Figure 4). As showed on the Fagan plot, the prior probability was 20%, posterior probabilities of PLR and NLR were 61% and 7%, respectively (Figure 5). In general, IOUS demonstrated high diagnostic accuracy in detecting glioma residues during surgical operation.

### Stratified analysis

In order to assess the impact of heterogeneity, we conducted subgroup analyses to further research the diagnostic power of IOUS in glioma residual resection. We suspected that study region, mean age, sample size and glioma grading could affect the pooled parameters of IOUS diagnosis evaluation. Further studies showed that parameters in mean age, sample size and region were no obvious difference between the pooled data, which suggesting those factors were not greatly impact on heterogeneity. However, the sensitivity of low-grade glioma reached a high accuracy (rose from 0.75 to 0.87). The meta-regression analysis revealed IOUS was more accurate for low-grade glioma than high-grade glioma to detect residual (AUC 0.93 vs. 0.83). But it limited to few samples, it will be more robust and credible with increased studies and standardized methodology. The detail parameters for each subgroup are summarized in Table 2.

**Table 2:** The pooled sensitivity, specificity, PLR, NLR and DOR, and their 95% CI for each subgroup

Category	SEN(95% CI)	SPE(95% CI)	PLR(95% CI)	NLR(95% CI)	DOR(95% CI)	AUC(95% CI)
**Region**						
Asian	0.73 [0.63–0.8]	0.89 [0.78–0.94]	6.43 [3.56–11.62]	0.30 [0.23–0.40]	21.23 [13.^20^–^34^.^15^]	0.86 [0.83–0.89]
Europe	0.66 [0.58–0.74]	0.82 [0.69–0.91]	3.59 [2.08–6.19]	0.21 [0.03–1.45]	19.82 [7.69–51.12]	0.88 [0.84–0.89]
**Mean age(y)**						
≤ 41.6	0.73 [0.60–0.83]	0.91 [0.78–0.96]	7.68 [3.47–16.99]	0.30 [0.21–3.87]	25.44 [13.52–47.87]	0.87 [0.84–0.90]
> 41.6	0.75 [0.50–0.90]	0.84 [0.68–0.93]	4.64 [2.76–7.78]	0.29 [0.14–0.60]	15.76 [8.90–27.91]	0.87 [0.83–0.90]
**Sample size**						
≤ 100	0.81 [0.59–0.93]	0.77 [0.64–0.86]	3.47 [2.46–4.88]	0.25 [0.12–0.54]	13.88 [6.71–28.73]	0.84 [0.81–0.87]
> 100	0.66 [0.58–0.73]	0.94 [0.86–0.97]	10.54 [5.06–21.95]	0.36 [0.30–0.44]	29.15 [15.14–56.15]	0.81 [0.77–0.84]
**Glioma grading**						
Low	0.87 [0.77–0.94]	0.89 [0.80–0.95]	4.71 [1.35–16.43]	0.20 [0.11–0.35]	45.37 [14.99–137.4]	0.93 [0.91–0.96]
high	0.76 [0.67–0.84]	0.75 [0.62–0.85]	2.84 [1.36–5.93]	0.31 [0.18–0.56]	10.43 [4.11–26.45]	0.83 [0.80–0.85]

### Sensitivity analysis and publication bias

**Figure 6:**
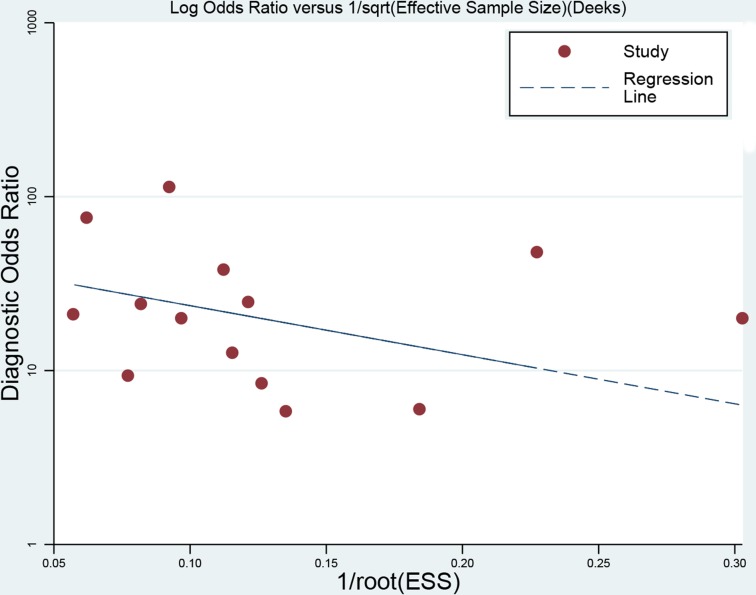
Deek's funnel plot to evaluate the publication bias

We performed sensitivity analysis by excluding individual study sequentially. There was no remarkable variation for pooled parameters (the detail data were not given), indicating the pooled estimations were stable. The Deek's plot were used to evaluate the publication, as the Figure 6 indicates that was no significant publication bias exist (t = −1.03, *P* = 0.33).

## DISCUSSION

Nowadays, more fully precise excision of glioma is a challenge for neurosurgeons, the large extent of tumor resection could influence the prognosis and quality of life for patients. Ultrasound is an old and common technology, but lack of sufficient evidence to prove that is a valid method tool at characterizing glioma residual. The high sensitivity and specificity (0.75 and 0.88) in this meta-analysis revealed that IOUS is a meritorious tool to distinguish glioma margins clearly in surgical excision, especially in low-grade glioma with larger AUC than high-grade glioma. Distinguished edema from irregular shape and infiltrating growth is probably hard for high-grade glioma, even ultrasound can display tumor areas at normal blood brain barriers [24].

An appropriate diagnostic method include accuracy, real-time, low price, and effectiveness. IOUS meets these requirements and can be performed multiple times [25]. According to Woydt's tumor residual norm [10], irregular hyperecho or annular strong echo stretches into the surrounding brain tissues of residual cavity with the thickness ≥ 5 mm as tumor residue. We used IOUS to detect glioma location, tumor size, the distance from cortex, surrounding blood supply and para-carcinoma tissues for identifying the extent of tumor removal. The results showed diagnostic odds ratio is 21.83 and the pooled PLRs are 6.27 and 0.29, which indicate the probability of tumor residues diagnosis compared with brain tissues is 6.27 times in case of hyperecho exceeds the cut-off value. On the contrary, the probability of definite case is 29% when it is below the cut-off value. IOUS has unique advantages, but it cannot fully replace other detection tools with the interference of ultrasonic pseudo morphism, bubbles, blood clot [26]. As we know, neuronavigation is more accurate for tumors less than 1 cm, but the application is restricted by brain deformation or shifting after skull opening [27–28]. So an integration of IOUS into neuronavigation could offer a combine image to determine the tumor boundary.

Now give some suggestion to avoid false positives by using proper operation and sufficient experience. (i) remove hemostasis material from the residual cavity before using ultrasound; (ii) using physiological saline to improve diagnostic accuracy; (iii) discern the echo size and position among tumor tissue, distinguish edema and colloid hyperplasia carefully; (iv) the hematoma and edema with clearer boundary presented stronger echo, but necrosis and cystic degeneration is hypoechoic. The examiner must be aware of ultrasound technology to define margins and assess the degree of resection in surgery.

In recent years, contrast enhanced ultrasound provides more anatomy and pathology information and offers clearly tumor boundary by new-type contrast agents appeared [29]. With the improvement of ultrasound technology, linear-array ultrasound has been offered to further resection in surgery operation. IOUS has become an indispensable tool to judge the degree of glioma resection, identify gliosis from gliomas and estimate tumor pathology classification [30].

In conclusion, this systematic review and meta-analysis was to evaluate the diagnostic features of intraoperative ultrasound, which has a significantly higher diagnosis accuracy. Current evidence suggests that IOUS may be a useful and non-invasive method for detecting glioma margin and allows safer removal.

## MATERIALS AND METHODS

### Search protocol

We conducted a literature search through following electronic databases: PubMed, Embase, Web of Science, CNKI, Wanfang and Weipu updated to May 5, 2017. Meanwhile, Google and Baidu engine were searched by comparing IOUS and pathological diagnostic criteria for treatment of glioma residues. Our research protocol consists of the detailed search strategy, screening criteria for titles, abstracts, and full-text articles. The search terms included “intraoperative ultrasound” or “ultrasonography” or “IOUS” and “intracranial glioma” or “glioma” and “residue” or “remnant”. In order to find all potentially eligible articles, we searched the reference lists manually for relevant study. The Preferred Reporting Items for Systematic Reviews was used in this meta-analysis [31]. The checklist guidelines was shown in Supplementary Table 1.

### Selection criteria

The inclusion criteria were as follows: 1) all cases were performed by IOUS before harvesting pathological results; 2) the process of IOUS was evaluated; 3) raw data were sufficient to calculate true positive (TP), false positive (FP), false negative (FN) and true negative (TN); 4) abstract, review, conference paper and case report (less than 5 cases) were excluded; 5) no partial data or repetitive publication was permitted; 6) intracranial tumors were not admitted (except intracranial glioma), such as cavernous hemangioma, metastatic encephala, and radiation necrosis; 7) only human studies were included and the language was limited in English and Chinese.

### Data extraction and quality assessment

According to the above criteria, two authors independently filtered the unqualified studies by reading the titles and abstracts, and then scanned full texts of the remaining articles to find the eligible one. Duplicate records were deleted. In this process, any divergence was resolved through discussion. The extracted data included article serial number, title, the name of first author, country, year of publication, mean age and sample size. Moreover, specificity, sensitivity and TP/FP/FN/TN were extracted from each eligible article or could calculate relevant data from original article.

The selected articles quality were assessed by the Quality Assessment of Diagnostic Accuracy Studies 2 (QUADAS-2) [32], which included two fields of risk of bias and applicability. Each field consists of four items: patient selection, index text, reference standard, and flow & timing. Each item contains 17 sub-clauses as questions. We scored the questions as “low”, “high”, or “unclear” to examine diagnostic studies.

### Statistical analysis

The pooled sensitivity, specificity, diagnostic odds ratios (DOR), positive likelihood ratios (PLR), negative likelihood ratios (NLR), area under the curve (AUC) and the corresponding 95% confidence intervals (CIs) were calculated by bivariate regression model on Stata 12.0 software and Review manager 5.3. The summary receiver operator characteristic (SROC) curves were plotted for graphical assessment. Threshold effect from different diagnostic cutoff values was tested by Spearman correlation analysis. Heterogeneity was detected via *Q* test and I^2^ statistic, with I^2^ > 50% and *P* < 0.05 indicating the presence of heterogeneity [33–34]. If heterogeneity existed, a random effects model was performed, otherwise a fixed effects model was used. And subgroup analysis was conducted to dissect the heterogeneity. Publication bias was evaluated visually by using Deek's funnel plots; the likelihood ratio, prior probability and posterior probability were tested by Fagan plot [35]. The *P* < 0.05 was considered as statistical significance.

## SUPPLEMENTARY TABLE AND FIGURES





## Abbreviation

IOUS: intraoperative ultrasound
TP: true positive
FP: false positive
FN: false negative
TN: true negative
QUADAS-2: Quality Assessment of Diagnostic Accuracy Studies
PLRs: positive likelihood ratios
NLRs: negative likelihood ratios
DORs: diagnostic odds ratios
SROC: summary receiver operator characteristic curve area
CI: confidence interval
