# Supplementary material for: Diagnostic ability of intraoperative ultrasound for identifying tumor residual in glioma surgery operation
Source: Oncotarget. 2017 Aug 22;8(42):73105–14. doi: 10.18632/oncotarget.20394 (PMC5641196; doi:10.18632/oncotarget.20394)
Supplement: Supplementary file 1 [file oncotarget-08-73105-s001.pdf]

Diagnostic ability of intraoperative ultrasound for identifying tumor residual in glioma surgery operation: a systematic review and meta-analysis

SUPPLEMENTARY MATERIALS

Supplementary Table 1: 2009 Checklist guidelines of this meta-analysis. See Supplementary_Table_1

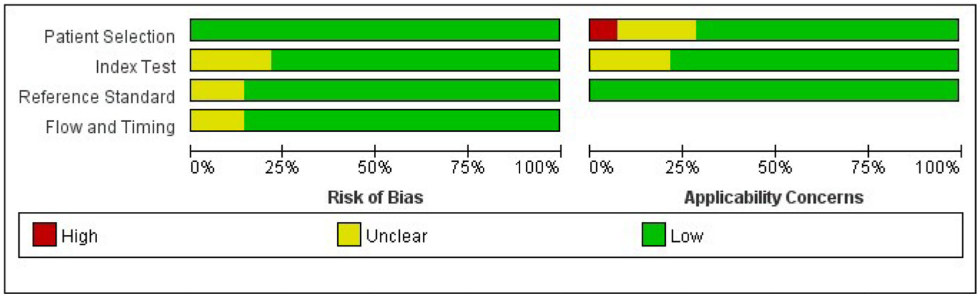

Supplementary Figure 1: Risk of bias and applicability concerns graph: review authors' judgement about each domain presented as percentages across included studies.

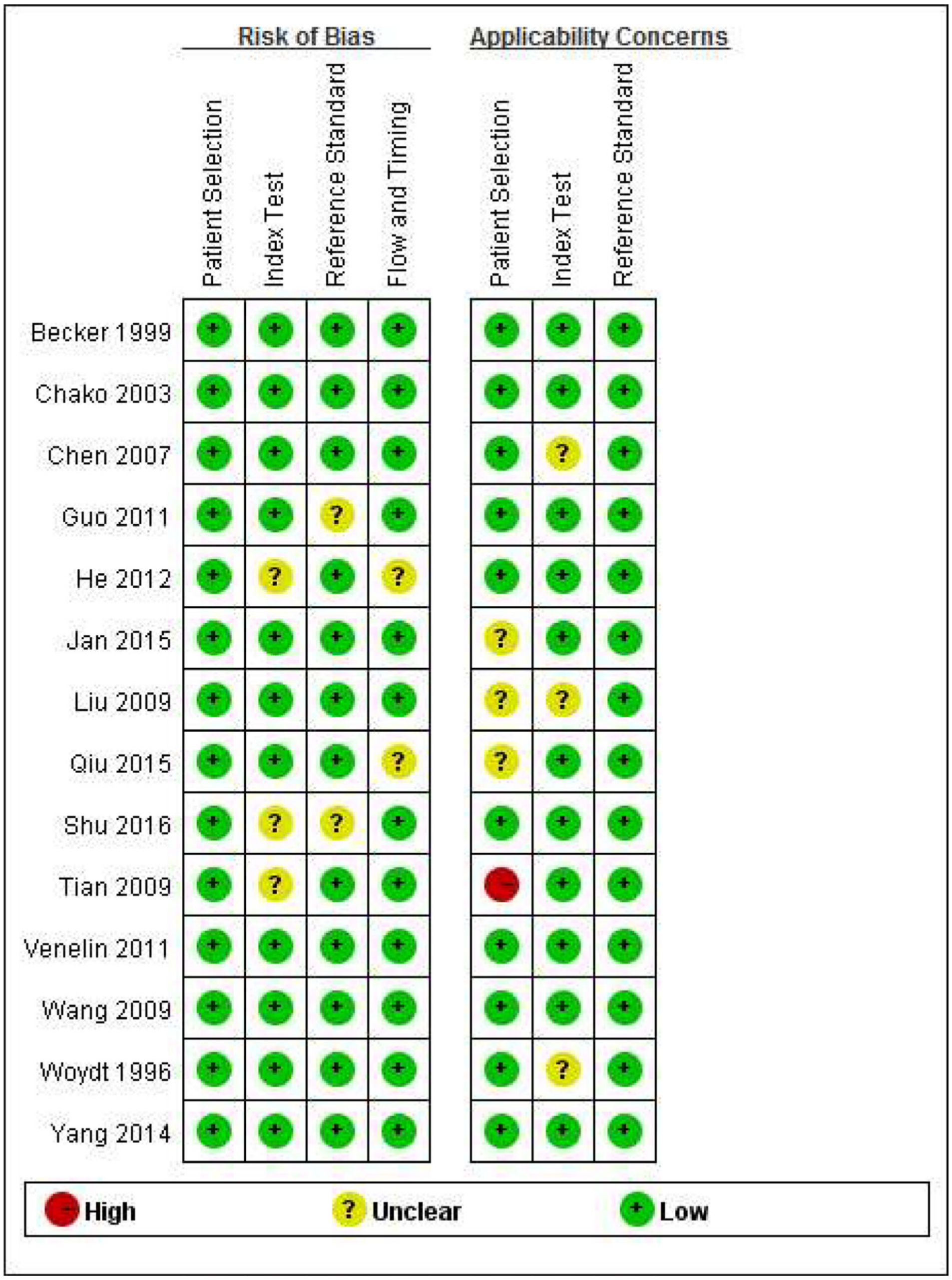

Supplementary Figure 2: Risk of bias and applicability concerns summary: review authors' judgement about each domain for each included study.
